# Influence of NaYF_4_ Inert and Active Layer on Upconversion Luminescence

**DOI:** 10.3390/nano12193288

**Published:** 2022-09-21

**Authors:** Zhaojin Wang, Shebao Lin, Yajun Liu, Jin Hou, Xinyi Xu, Xin Zhao, Biying Wei

**Affiliations:** Institute of Physics and Optoelectronics Technology, Baoji University of Arts and Sciences, Baoji 721016, China

**Keywords:** rare earth luminescent nanomaterials, upconversion emission, energy transfer, synthesis of luminescent nanoparticles

## Abstract

NaYF_4_:Yb,Er@NaYF_4_ core–shell nanostructures were prepared to investigate their influence on upconversion (UC) luminescence. Tests revealed green radiation (^4^S_3/2_→^4^I_15/2_) and red radiation (^4^F_9/2_→^4^I_15/2_) first increased and then gradually decreased as Yb concentration increased in the NaYF_4_ shell. The strongest fluorescent radiation occurred at an Yb concentration of 5%. To investigate the complicated variation of luminescence, we designed a set of experiments to study the impact of Yb ion concentration on luminescence intensity, and we analyzed the corresponding enhancement mechanism. It is probable that the energy transfers between both Yb and Er ions and Yb and Yb ions are involved in the UC processes. The enhancement of hybrid nanostructures has huge potential in biological detection and solar cells.

## 1. Introduction

Lanthanide-doped upconverting nanoparticles (UCNPs) have attracted much attention due to their application in laser materials, solar cells, biological imaging, and display technologies [1,2,3,4,5,6]. Nevertheless, the absorption coefficients of lanthanide ions are small, resulting in low fluorescence emission efficiency of the UCNPs [7,8,9,10]. The approaches that are adopted to improve luminescence efficiency include introducing core–shell nanostructures [11,12,13,14,15,16]. An inert layer increases radiant efficiency by repairing defects on the surface of UCNPs, whilst the active layer transfers more energy to the activation ions in the nuclear region. For example, Yi et al. reported that the UC emission intensity was enhanced by 7.4 times in hexagonal phase NaYF_4_ NPs through coating with a 2 nm NaYF_4_ thick layer [17]. Meanwhile, heterogeneous NaYF_4_:Yb^3+^/Er^3+^@NaGdF_4_ core–shell (CS) structures with magnetic resonance imaging (MRI) properties were synthesized [11]. In addition, Wang et al. synthesized NaYF_4_:Ln^3+^@CaF_2_ core–shell nanoparticles and obtained a 300-fold enhancement, and the effect of the thickness of CaF_2_ on UC luminescence was also investigated [18]. The study found that a coated shell can effectively enhance the luminescent properties of the material, which is due to the shell being able to isolate the energy transfer between activated ions and surface organic groups. Gong et al. reported that the UC emission was enhanced by 21 times after active layer coating [19]. When coated with an active layer, the excited Yb ions in the shell can transfer energy to the surrounding Yb ions, and the Yb ions in the shell also can transfer energy to Yb ions or Er ions in the core. This process can effectively increase the acquisition of the excitation energy of the luminescent center. However, the influence of Yb ion doping concentration in the shell on upconversion emission and the concentration quenching effect will be enhanced. Therefore, exploring a suitable excitation concentration of Yb ions in the NaYF_4_:Ln^3+^@NaYF_4_:Yb^3+^CS UCNPs becomes critical for researchers.

In this research, an attempt was made to enhance the UC emission intensity of NaYF_4_:Yb,Er NPs by building a core–shell structure and employing doping with different Yb concentrations. The impact of the NaYF_4_ inert layer on UC emission and an optimal Yb ion doping concentration were investigated. The relationship between Yb ion doping concentration and spectral correlation was studied as well. It is hoped that this approach will achieve stronger upconversion emission and provide a new experimental basis for further expanding its application in biomedical imaging, anti-counterfeiting, and color display.

## 2. Experimental Section

### 2.1. Materials

Sinopharm Co. (Shanghai, China) provided YCl_3_•xH_2_O, YbCl_3_•xH_2_O, ErCl_3_•xH_2_O, NH_4_F, NaOH, CH_3_CH_2_OH, CH_3_OH, cyclohexane, oleic acid(OA), octadecene(ODE), ammonia solution(NH_3_•H_2_O), and Na_3_C_6_H_5_O_7_•2H_2_O of analytical grade. All chemicals were used as supplied.

### 2.2. Synthesis of NaYF_4_:Yb,Er Core Nanoparticles

NaYF_4_:Yb,Er NPs were prepared by the high-temperature coprecipitation way as described in detail in the literature [20]. First, 0.78 mM YCl_3_, 0.20 mM YbCl_3_, and 0.02 mM ErCl_3_ were added to a 100 mL three-necked bottle. The three-necked bottle contained 6 mL of oleic acid (OA) and 15 mL octadecene (ODE). The chemicals were mixed thoroughly, and the mixture was allowed to react at 160 °C for half an hour; then, it was cooled to 50 °C, and 10 mL of methanol mixture including 2.5 mm NaOH and 4 mm NH_4_F was added and fully stirred for 30 min. The solution temperature was elevated and kept at 100 °C for 15 min. The methanol was allowed to evaporate at high temperature, and the temperature was increased to 300 °C; the reaction solution was protected with inert gas (Ar) for one hour in the meantime. The solution was naturally cooled to room temperature. Ethanol was added and mixed to separate the sample particles. The particles were collected and precipitated for 5 min at high speed. The particles were centrifuged several times at high speed in ethanol to obtain clean nanoparticles and then transferred to 6 mL cyclohexane for preservation.

### 2.3. Synthesis of NaYF_4_:Yb,Er@NaYF_4_ Core–Shell Nanoparticles

Following the synthesis process in [21], 0.2 mM YCl_3_, 3 mL OA, and 7.5 mL ODE were first mixed in a 100 mL three-necked flask and heated at 160 °C for 30 min. The mixture was heated at 160 °C for 30 min. Then, 50 mg NaYF_4_:Yb,Er core NPs dispersed in 5 mL cyclohexane were added, and 5 mL methanol solution including 2.5 mM NaOH and 4 mM NH_4_F was added. The mixture was stirred for 30 min at 50 °C. Different amounts of YCl_3_, NaOH, and NH_4_F were used to prepare NaYF_4_ shells of different thicknesses. Subsequently, the solution was heated to 100 °C for 15 min. Methanol and cyclohexane were allowed to evaporate at high temperature, and the temperature was increased to 300 °C; the reaction solution was allowed to stand in an inert gas (Ar) for one hour. The particles were collected and precipitated for 5 min at high speed after the solution was cooled naturally to 50 °C. The particles were centrifuged several times at high speed in ethanol to obtain clean nanoparticles and then transferred to 6 mL cyclohexane for preservation.

### 2.4. Characterization of Samples and Measurement of Spectra

Powder X-ray diffraction (XRD) patterns were determined using a D/Max2550 diffractometer (Rigaku Inc., Tokyo, Japan). The microscopic morphology of the samples was measured and characterized using a transmission electron microscope (TEM, JEOL 2100, Tokyo, Japan) at 200 kV. Luminescence characteristics were measured by analyzing spectra. A titanium sapphire femtosecond laser and a 980 nm diode laser were used as excitation sources for spectral measurements. We collected and detected luminescence with a spectrometer (SP2750i, Princeton Inc., Trenton, NJ, USA). Lifetime was measured using a photomultiplier tube and a Boxcar. All measurements of the luminescence spectrum were performed at room temperature.

## 3. Results and Discussion

### 3.1. Sample Crystal Phase and Sample Morphology 

**Figure 1:**
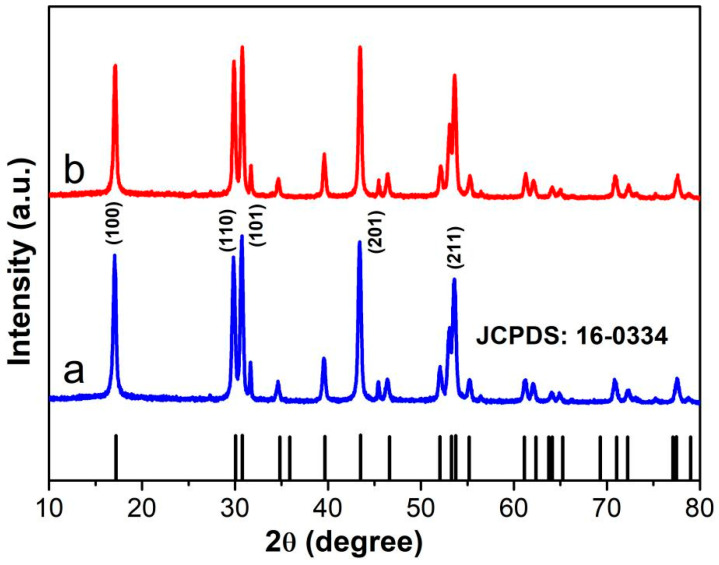
XRD mappings of (a) NaYF_4_:Yb^3+^/Er^3+^ and (b) NaYF_4_:Yb^3+^/Er^3+^@NaYF_4_:Yb^3+^ nanocrystals.

The microstructure and crystal structure of the finished products were characterized using XRD and TEM. Figure 1 shows that the XRD patterns of NaYF_4_:Yb,Er and NaYF_4_:Yb,Er@NaYF_4_ samples are pure hexagonal NaYF_4_ (JCPDS 16-0334).

**Figure 2:**
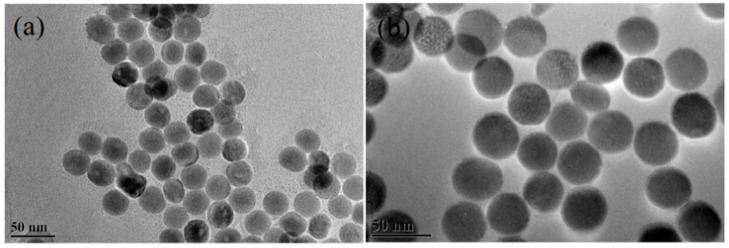
TEM pictures of (**a**) NaYF_4_:Yb^3+^/Er^3+^ and (**b**) NaYF_4_:Yb^3+^/Er^3+^@NaYF_4_:Yb^3+^ nanocrystals.

TEM measurements were undertaken to characterize the morphology of the sample shown in Figure 2. It was concluded that UCNPs showed excellent homogeneity and monodispersity. The mean diameter of the NaYF_4_ sample measured about 27 nm. In the NaYF_4_:Yb,Er@NaYF_4_ core–shell nanoparticles, the NaYF_4_ layer was uniformly coated around NaYF_4_:Yb,Er particles, as shown in Figure 2b. The NaYF_4_:Yb,Er@NaYF_4_ had a mean diameter of about 35 nm. In this investigation, the shell measured about 4 nm.

### 3.2. Effect of Inert Shell on Fluorescence

**Figure 3:**
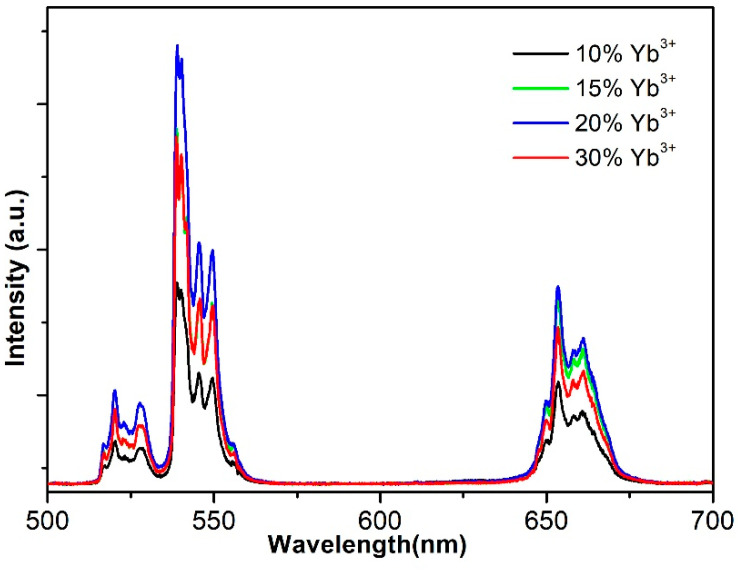
The UC emission spectrum of NaYF_4_:x%Yb^3+^,2%Er^3+^.

The luminescence of NaYF_4_:Yb,Er with 980 nm laser excitation was explored. Figure 3 shows the corresponding UC emission spectrum. The spectra of the samples reveal two sharp emission peaks due to the ^4^S_3/2_→^4^I_15/2_ and ^4^F_9/2_→^4^I_15/2_ [22] energy level transition. The UC emission intensity shows a pronounced change with different Yb concentrations, as shown in Figure 3. The emission intensity amplitude of the green light and red light strengthened gradually as the concentration of Yb increased. When the concentration of Yb reached 20%, the fluorescence intensity was at its maximum, and when the concentration of Yb continued to increase to 30%, the fluorescence intensity decreased noticeably. Thus, 20% is the optimal Yb ion doping concentration. The above phenomenon indicates that if Yb ion concentration is low and increasing, energy transfer efficiency from Yb ions to Er ions is enhanced. However, when the Yb ion concentration exceeds 20%, the distance between Yb ions decreases, and the energy transfer efficiency between Yb ion pairs increases [23], thus reducing the energy transfer efficiency from Yb ions to Er ions.

**Figure 4:**
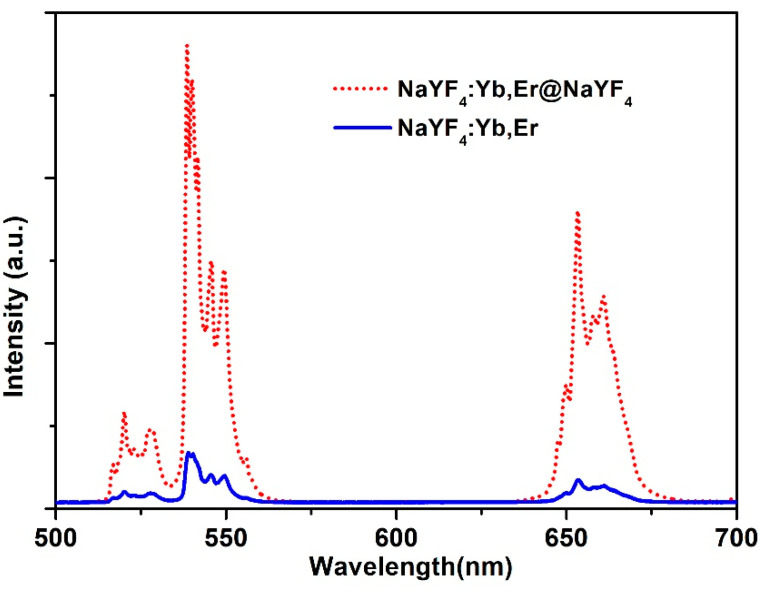
The UC emission spectrum of NaYF_4_:20%Yb^3+^,2%Er^3+^ and NaYF_4_:20%Yb^3+^,2%Er^3+^@NaYF_4_ nanocrystals. Excitation wavelength is 980 nm.

Figure 4 shows the UC emission spectrum of NaYF_4_:Yb,Er and NaYF_4_:Yb,Er@NaYF_4_ NPs under 980 nm excitation. The UC emission intensity of NaYF_4_:Yb,Er NPs was greatly enhanced by coating the NaYF_4_ inert shell. Since there were no ions in the shell, we surmised that there were many surface defects and organic groups on the surface of the nanocrystals. These surface defects and the presence of organic groups may induce more radiation-free relaxation channels leading to reduced fluorescence emission [23]. It was noted that not only the Er ions near the surface but also those inside were affected. The inert shell can effectively repair the surface flaws of the nanocrystals and isolate the transfer of energy between the Er ions on the shell surface and the organic groups of the surface coating layer.

**Figure 5:**
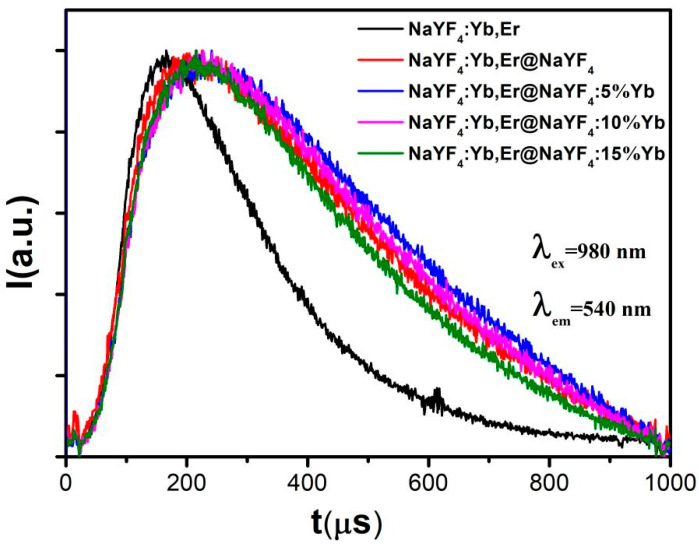
Energy level lifetime diagram of 540 nm from NaYF_4_:Yb^3+^,Er^3+^ and NaYF_4_:Yb,Er@NaYF_4_:x%Yb.

To verify the above analysis of changes in UC emission intensity, we carried out a UC emission dynamics test in which a 980 nm pulsed laser was selected as the excitation source. Figure 5 shows the UC emission attenuation curve at 540 nm of Er ions in NaYF_4_:Yb,Er and NaYF_4_:Yb,Er@NaYF_4_ samples. From the corresponding time decay curve, the rising edges and falling edges are clearly observed. The decay lifetime at 540 nm can be obtained by fitting the curve with a second-order exponential function. The average lifetime is

(1) $$\tau =\frac{A_{1}^{2}\tau_{1}^{2}+A_{2}^{2}\tau_{2}^{2}}{A_{1}\tau_{1}+A_{2}\tau_{2}}$$

A_1_ and A_2_ are constants; $\tau_{1}$ and $\tau_{2}$ are the corresponding decay times. The corresponding lifetimes of samples NaYF_4_:Yb,Er and NaYF_4_:Yb,Er@NaYF_4_ were 219 and 641 μs, respectively. According to the decay lifetime definition:

(2) $$\tau =\frac{1}{\Gamma_{\mathrm{rad}}+k_{\mathrm{nr}}},$$

τ is lifetime, $\Gamma_{\mathrm{rad}}$ is the radiative transition probability, and *k*_nr_ is the probability of non-radiative transition. According to the fitting results, the decay lifetime of 540 nm increases after coating the NaYF_4_ shell, and the radiative probability is considered unchanged. It can be concluded that the radiation-free probability decreases [24].

### 3.3. Effect of Active Shell on Fluorescence

**Figure 6:**
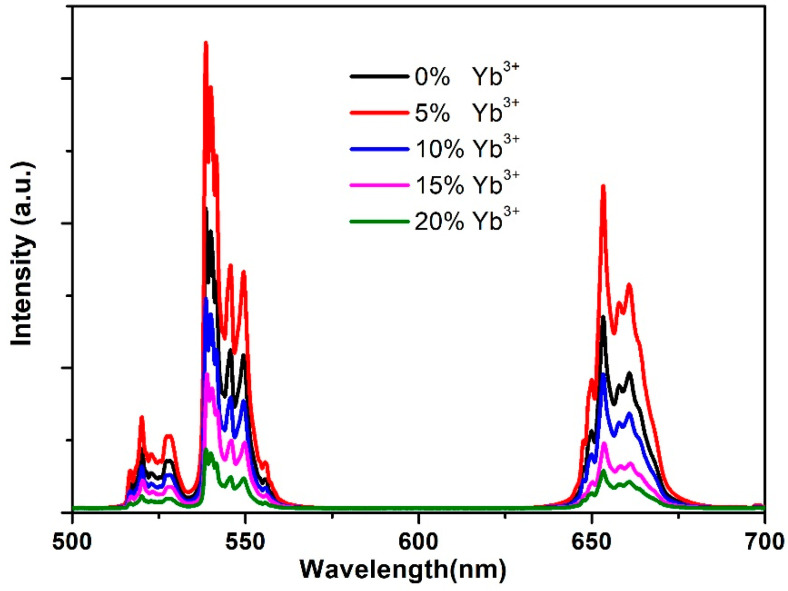
The UC emission spectrum of NaYF_4_:Yb^3+^,Er^3+^@NaYF_4_:x%Yb^3+^ nanocrystals with different Yb^3+^ concentrations.

Figure 6 shows the UC emission intensity distribution for NaYF_4_:Yb,Er@NaYF_4_:xYb NPs. It can be seen that the UC emission intensity varies with the coating layer. The emission intensity amplitude of the green and red gradually increases as the concentration of Yb increases; when the concentration of Yb ions is at 5%, UC emission intensity reaches its maximum, and when the concentration of Yb ions continues to increase to 20%, the fluorescence intensity decreases discernably. The optimal Yb ion doping concentration is 5%.

**Figure 7:**
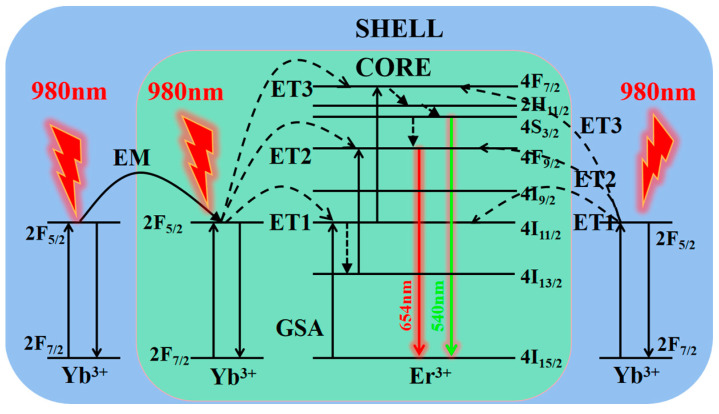
Energy level diagrams of Er^3+^ and Yb^3+^ ions as well as proposed UC mechanisms.

Figure 7 shows the energy level structures of Er and Yb ions and the corresponding upconversion luminescence processes. The Yb ions in the shell absorb the excitation light energy and then transmit it to Er and Yb ions in the core. Er ions in the core transition to the corresponding higher energy levels through non-radiation energy transfer, radiating green light and red light, etc.

As can be seen from Table 1, with the Yb ion doping concentration in the active shell increasing from 0 to 15%, the UC emission (540 nm) lifetime increases, reaches the maximum at 5%, and then gradually decreases. From 0 to 5%, Yb ions in the shell positively transfer energy to the ions in the core, and their luminescence is enhanced. After 5%, as the doping concentration of Yb ions increases, a reverse transfer process will occur, and high-level ions in the core will inversely transfer energy to Yb ions in the ground state of the shell, weakening the luminescence of Er ions in the core. The crossing relaxation between the Yb ions in the shell is increased, which leads to the increase in non-radiation probability and the decrease in UC emission lifetime.

To confirm our analysis of the changes in UC emission intensity, NaYF_4_:20%Yb,2%Er@NaYF_4_(A) and NaYF_4_:2%Er@NaYF_4_:20%Yb(B) were designed.

**Figure 8:**
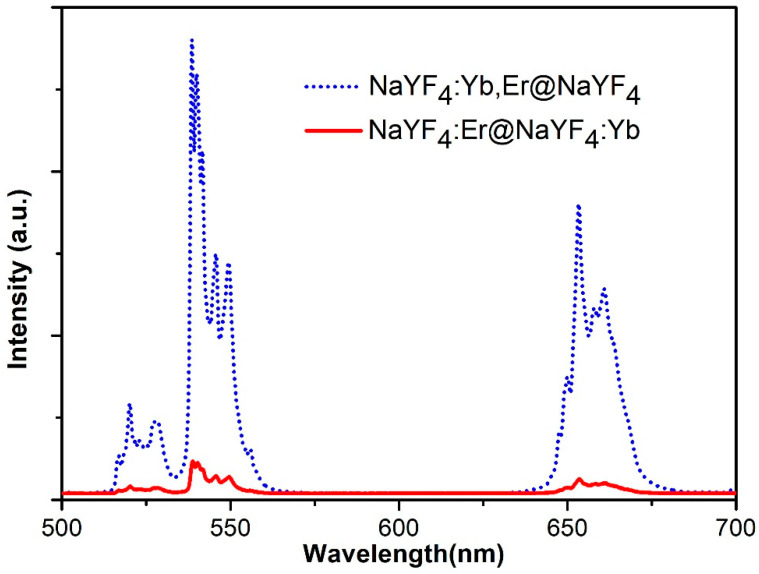
The UC emission spectrum of NaYF_4_:20%Yb^3+^,2%Er^3+^@NaYF_4_:Yb^3+^ and NaYF_4_:2%Er^3+^@NaYF_4_:20%Yb^3+^ nanocrystals.

Figure 8 shows the fluorescence intensity distribution of these two rare earth nanoparticles with core–shell structures. The excitation conditions are identical, with the excitation wavelength at 980 nm. The upconversion fluorescence intensity of sample A is much stronger than that of sample B, while the excitation power and ion quantity of the two samples remain unchanged. However, such a significant difference in UC emission intensity indicates that the energy transmission efficiency from Yb ions in the shell to the Er ions in the core is quite different from that of the ions in the core. The energy transmission efficiency from Yb ions in the shell to Er ions in the core is much lower than that of Yb ions to Er ions in the core.

It is generally believed that the fluorescence intensity of the core–shell nanostructure reaches the maximum when the shell Yb concentration is 20%. In fact, when the shell Yb concentration is 20%, the UC emission intensity of core–shell nanoparticles is at its lowest.

The analysis indicates that the energy transmission efficiency of Yb ions in the core to Er ions in the shell is far lower than that of Yb ions to Er ions in the same core, and the absence of Er ions in the shell will inevitably weaken the positive factors. Due to the absence of activated ions, energy migration between the excited Yb ions in the shell is more efficient, which undoubtedly reinforces the negative factor. Thus, the above qualitative analysis shows that the optimal shell Yb ion doping concentration should be lower than 20%.

More energy is transferred to the active ions when the Yb ion concentration in the shell increases. This is a positive factor in enhancing upconversion emission. On the other hand, the increase in the concentration of Yb ions will decrease the distance between Yb ions in the shell. This makes the energy transfer between Yb ions easier and thus readily transfers the absorbed energy to the defects on the shell surface. This also reduces the fluorescence quantum efficiency, a negative factor for enhancing UC emission intensity. Therefore, an optimal Yb ion doping concentration exists to obtain the best luminescence, which is the equilibrium position of the two competing processes. The experiment shows that UC emission intensity is the strongest when the concentration of Yb ions is 5%.

## 4. Conclusions

NaYF_4_:Yb,Er@NaYF_4_:xYb core–shell structures have been synthesized, and their UC emissions have been investigated. The results show that the green and red UC emission increases first and then decreases gradually as the concentration of Yb increases. When the Yb ion concentration is 5%, UC emission intensity is at its maximum, and when it continues to increase to 20%, UC emission intensity clearly decreases. The optimal Yb ion doping concentration is 5%.

Two energy transfer paths exist after the Yb ions absorb the excited light energy. When the concentration of Yb ions is low, energy is transferred from the excited-state Yb ions to the nearby Er ions, and when the concentration of Yb ions is high, energy is transferred from the excited-state Yb ions to the surrounding ground-state Yb ions. Therefore, UC emission intensity reaches the maximum when the Yb ion concentration is 5%.

## Figures and Tables

**Table 1:** Calculated lifetimes of Er^3+^.

Sample	Lifetime (μs) 540 nm
NaYF_4_:Yb,Er	219.7
NaYF_4_:Yb,Er@NaYF_4_	641.2
NaYF_4_:Yb,Er@NaYF_4_:5%Yb	1205.1
NaYF_4_:Yb,Er@NaYF_4_:10%Yb	740.1
NaYF_4_:Yb,Er@NaYF_4_:15%Yb	573.7

## Data Availability

The data presented in this study are available on request from the corresponding author.
